# Klotho, Aging, and the Failing Kidney

**DOI:** 10.3389/fendo.2020.00560

**Published:** 2020-08-27

**Authors:** Sarah Buchanan, Emilie Combet, Peter Stenvinkel, Paul G. Shiels

**Affiliations:** ^1^Wolfson Wohl Cancer Research Centre, Institute of Cancer Sciences, University of Glasgow, Glasgow, United Kingdom; ^2^School of Medicine, Dentistry & Nursing, Human Nutrition, Glasgow Royal Infirmary, Glasgow, United Kingdom; ^3^Division of Renal Medicine M99, Department of Clinical Science, Intervention and Technology, Karolinska Institutet, Stockholm, Sweden

**Keywords:** Klotho, aging, kidney, phosphate, senotherapeutic

## Abstract

Klotho has been recognized as a gene involved in the aging process in mammals for over 30 years, where it regulates phosphate homeostasis and the activity of members of the fibroblast growth factor (FGF) family. The α-Klotho protein is the receptor for Fibroblast Growth Factor-23 (FGF23), regulating phosphate homeostasis and vitamin D metabolism. Phosphate toxicity is a hallmark of mammalian aging and correlates with diminution of Klotho levels with increasing age. As such, modulation of Klotho activity is an attractive target for therapeutic intervention in the diseasome of aging; in particular for chronic kidney disease (CKD), where Klotho has been implicated directly in the pathophysiology. A range of senotherapeutic strategies have been developed to directly or indirectly influence Klotho expression, with varying degrees of success. These include administration of exogenous Klotho, synthetic and natural Klotho agonists and indirect approaches, via modulation of the foodome and the gut microbiota. All these approaches have significant potential to mitigate loss of physiological function and resilience accompanying old age and to improve outcomes within the diseasome of aging.

## Introduction

In 1997 Kuro-o et al. (1) reported a mutant mouse displaying a phenotype similar to that of premature aging in humans. The phenotype of shortened lifespan, multiple organ degeneration, vascular calcification (VC), cardio-vascular disease (CVD) and frailty were attributed to the abolished expression of the *klotho* gene, named after the Greek goddess of fate, κλωθ′ω, who spun the thread of life (1). Subsequent murine models, deficient in Klotho, displayed both an accelerated aging phenotype and abnormal phosphate and mineral regulation (2, 3).

Klotho has subsequently been characterized as one of a family of related proteins. These are all single-pass transmembrane proteins that include α-, β-, and ⋎- Klotho isoforms (3–5), the latter two discovered based on their homology with α-klotho (6, 7). β -Klotho is mainly expressed in the liver, but is also found in the kidney, gut and spleen. It regulates the activity of members of the fibroblast growth factor (FGF) family, including FGF-21 and FGF-19. ⋎-Klotho is expressed in the skin and the kidney and has yet to be ascribed defined functions (3, 7). α-Klotho comprises five exons and structurally its cognate protein is composed of a large extracellular domain followed by a transmembrane domain and a small domain of 11 residues comprising the intracellular C-terminus (8, 9). The extracellular domain comprises two repeat sequences termed KL1 and Kl2 which are generated by full-length transcript splicing and can be cleaved by the metalloproteases ADAM-10 and ADAM-17. Cleavage of the extracellular domain results in a soluble form of Klotho being released (8). Soluble Klotho is the main functional form in the circulation (10, 11) and is detected in the blood, urine, and cerebrospinal fluid (11–14).

Following the discovery of *fgf23* deficient mice displaying an identical aging phenotype to Klotho deficient mice, the Klotho protein was functionally identified as a receptor for Fibroblast Growth Factor-23 (FGF23) (2). FGF23 is a bone-derived hormone with a role in regulating phosphate excretion, which negatively regulates Vitamin D levels (15). Klotho is required for high affinity binding of FGF23 to its receptor FGFR1 (16); it is the resultant binary complex that constitutes the physiological receptor for FGF23 (17).

The Klotho protein that was identified as the FGF23 co-receptor was the α-Klotho isoform, which hereafter is termed Klotho. Klotho is expressed mainly on the cell surface membrane of proximal and distal renal tubules (18–20), though it is also found in the choroid plexus in the brain (21).

## Why Klotho Is Important in Aging

Klotho regulates many pathways involved in aging processes, such as the regulation of phosphate homeostasis, insulin signaling and Wnt signaling (5, 22). Additionally, Klotho also affects intracellular signaling pathways including p53/p21, cAMP, protein kinase C (PKC) and TGF-β (23, 24).

Klotho expression levels and its circulating level decline during aging. In humans, Klotho deficiency features medial calcification, intima hyperplasia, endothelial dysfunction, arterial stiffening, hypertension, impaired angiogenesis, and vasculogenesis (i.e., characteristics of early vascular aging) (25).

As Klotho-deficient phenotypes have been attenuated and rescued by Klotho gene expression, or supplementation, it is suggestive that Klotho has a protective effect with regard to the vasculature (26).

Mice deficient in Klotho exhibit a phosphate imbalance and hyperphosphatemia due to impaired urinary phosphate excretion, but significantly develop a complex progeric phenotype including poor growth, atrophy of multiple organs, vascular calcification (VC), sarcopenia, cardiac hypertrophy and fibrosis, cognitive impairment, and shortened lifespan (1, 2, 5). Consequently, Klotho knock-out mice display severe vascular disease, with widespread VC, endothelial dysfunction, and progressive atherosclerosis accompanied by hypervitaminosis D, hypercalcemia, and hyperphosphatemia (27).

As Klotho has been regarded as an anti-aging gene, in order to more fully understand how Klotho might impact age-related disease, this review focuses on what we actually mean by the aging process, its relevance to chronic kidney disease (CKD) as a disease of accelerated aging, and how this is impacted by Klotho. Furthermore, we discuss how modulation of Klotho might be exploited to mitigate the effects of the diseasome of aging.

### Aging

Rather than simply being a collection of morbidities arising during the final decades of life, aging is an active process across the entire life-course. It has been described as an accumulation of physiological and molecular deficits accruing at different rates, both within different tissues and organs in the same individual and between different individuals (28). Aging leads to a segmental and progressive loss of physiological function and physical capability over time, resulting in relative physiological frailty and loss of resilience (29–33). It is regulated actively by distinct biochemical pathways and has been characterized by a series of molecular and cellular hallmarks, which are common across taxa (33). These hallmarks comprise genomic instability, telomere attrition, epigenetic dysregulation, loss of proteostasis, dysregulated nutrient sensing, mitochondrial dysfunction, cellular senescence, with an accompanying senescence associated secretory phenotype (SASP), stem cell exhaustion, and altered intercellular communication (33).

### Aging and Phosphate—FGF23-Klotho Axis

In mammals, aging has a number of distinct additional features to its common hallmarks, comprising phosphate toxicity, diminished global Nrf2 expression, and microbial dysbiosis (34). These features are inherent in an associated “diseasome of aging,” where dysregulated aging processes are a common underpinning feature of individual morbidities. These reflect an individual's “burden of lifestyle” and the generation of allostatic (over)load at a molecular and cellular level, with concomitant loss of physiological resilience and capability (34, 35). It is within this framework that phosphate makes a significant contribution and why its interaction with Klotho is important.

Serum phosphate (Pi) levels shows an exceptionally strong negative correlation with lifespan in mammals (32, 36), Phosphate metabolism and reabsorption by the kidney is regulated via interaction between the kidney, bone, and gut, controlled by a cross-regulating endocrine network comprising para-thyroid hormone (PTH), vitamin D, FGF-23, and Klotho (5). As mice lacking α-Klotho, or FGF23, show phosphate retention (37), this suggests that this signaling axis is essential for Pi homeostasis. Several studies have shown that FGF23 increases urinary excretion of Pi and indirectly suppresses intestinal Pi absorption via down-regulation of vitamin D3 (15, 38, 39). Unsurprisingly, both FGF23 and Klotho deficient mice display a progeric phenotype and associated abnormal Pi regulation (2, 3). Additionally, Klotho deficient mice also display high FGF23 levels (40), and as a consequence of loss of Klotho expression may result in maladaptive signaling downstream of the FGF-receptor (FGFR) that may contribute to CKD associated co-morbidities, in particular cardiovascular co-morbidity. For example, this may result in a switch in FGF23-induced signaling in cells expressing FGFR4, but not α-Klotho (40). Critically, in support of this thesis, specific FGFR4 blocking antibodies have been demonstrated to attenuate left ventricular hypertrophy (LVH) in 5/6 nephrectomized rats, emphasizing that FGFR4 activation is a critical patho-mechanistic feature underlying LVH in states of α-Klotho deficiency and elevated levels of FGF23 excess (41).

Pertinent to these observations are observations of Klotho signaling independent of FGFR4 in the heart, liver, and lung (40, 42). In the kidney context dependent signaling has also been reported; neither FGFR3 nor FGFR4 is the principal mediator of FGF23 effects in the proximal tubule, but co-localization of FGFR1 and Klotho indicates that the effector site for FGF23 may be in the distal tubule (43). A reduction in tubular phosphate reabsorption may enable hyperphosphatemia to occur at earlier stages of CKD.

**Figure 1:**
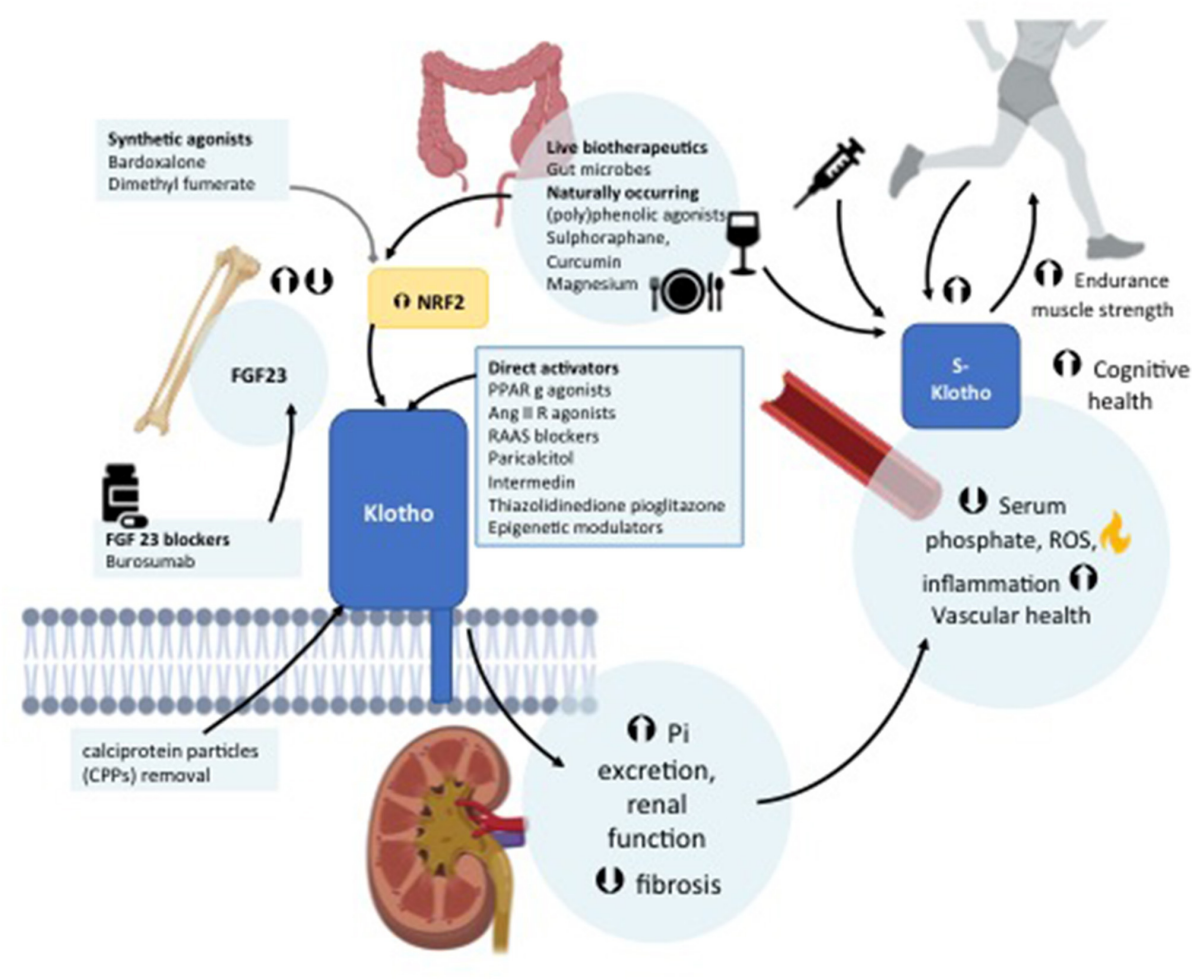
Schematic representation of the role of Klotho in the regulation of bone and mineral metabolism.

It is unclear how changes in serum Pi are detected, however, it is thought to involve sensing of calciprotein particles (CPPs), which are colloids comprised of calcium-Pi bound to fetuin A, a circulating inhibitor of vascular calcification (5, 37, 44). In proximal tubules, blood-borne FGF23 binds to α-Klotho-FGF receptors 1c, 3c, and 4 complexes, directly activating ERK 1/2 and SGK-1 signaling cascades, leading to down-regulation of the Na/H exchange regulatory cofactor NHERF-1 expression, localized to the brush border membrane of proximal tubular cells, a major site of Pi reabsorption (38). Down-regulation of NTP2A results in decreased Pi reabsorption in proximal tubules and increased urinary Pi excretion (8, 45). Active vitamin D and PTH both induce FGF23 expression (46, 47). Vitamin D has been shown to induce receptor hetero-dimerization with the retinoid receptor, resulting in the up-regulation of vitamin D responsive element target genes including FGF23 (48). PTH binding to its receptor results in PKA activation and the suppression of sclerostin, antagonizing the WNT signaling pathway and the suppression FGF23 expression (49). Serum levels of both active vitamin D and PTH are both reduced by FGF23, whilst FGF23 suppresses the synthesis of active vitamin D by up-regulation of *Cyp24a1*, which encodes 24-hydroxylase, responsible for the conversion to inactive vitamin D by a Klotho dependent mechanism (Figure 1). *Cy27b1* expression, which converts the D3 form to the active form of vitamin D, is downregulated. PTH is suppressed via both α-Klotho dependent and independent pathways. α-Klotho is expressed in the parathyroid gland and FGF23 suppresses both the expression and secretion of PTH (46). Serum levels of FGF23 correlates with both serum Pi and calcium levels and evidence suggests that both Pi and calcium are required to stimulate FGF23 levels by osteocytes (50, 51). Notably, hyperphosphatemia has been shown to induce DNA damage in vascular smooth muscle cells, resulting in cellular senescence, which further contributes to the premature aging process observed with Klotho dysregulation or loss (3, 52). This has implications for clinical conditions where Klotho may play a role. This is now discussed below with specific reference to CKD as a disease of aging.

### CKD and Aging

CKD is part of the diseasome of aging and displays an overlapping phenotype with Klotho-deficient mice. It has been suggested that Klotho function is tightly correlated with the pathogenic mechanisms underpinning CKD (18, 53–55).

CKD is a progressive systemic disease that affects 10–12% of the population (56). The prevalence of CKD has increased markedly over the past decades due to an aging population worldwide and an increase in incidence of obesity and type-2 diabetes; a significant driver of CKD (57). Progressive kidney damage leads to an increased number of systemic complications, hallmarked by persistent (chronic) low-grade inflammation, including hyperphosphatemia, CVD, anemia, hypertension, mineral-bone disorders, muscle wasting, osteoporosis, increasing frailty, and anemia (58). In earlier stages of CKD, a reduction in the reabsorption of Pi by the kidney triggers increased FGF-23 secretion that promotes renal Pi secretion. However, when patients reach CKD stage 3 systemic hyperphosphatemia usually occurs, which contributes to systemic inflammation and VC leading to early vascular aging (EVA) (59). Hyperphosphatemia promotes endothelial dysfunction (60) and induces expression of the pro-inflammatory NFkB pathway (61). Impaired kidney function results in the accumulation of systemic nitrogenous compounds which leads to persistent inflammation involving both immune activation and immunosuppression (62). The uremic phenotype within CKD is characterized by an increased frequency of associated age-related complications, such as vascular stiffening, osteoporosis, muscle wasting, depression, cognitive dysfunction, and frailty (58, 63). A model of four key mechanisms has been proposed for the pathogenesis of premature aging based upon: (i) a persistent increase in allostatic load owing to oxidative stress, inflammation (inflammageing), and disturbances in sympathetic-vagal balance and circadian rhythm; (ii) activation of the stress resistance response which contributes to inhibition of anabolic pathways, increased protein catabolism, and tissue atrophy; (iii) disease-associated alterations that directly promote the aging process; (iv) defective anti-aging mechanisms (58). If allostatic response mechanisms remain chronically activated, systemic complications then follow.

Chronic low-grade inflammation also contributes to the premature aging phenotype in CKD (64) and other “burden of lifestyle” diseases, such as chronic obstructive pulmonary disease (COPD), chronic heart failure (CHF), and rheumatoid arthritis (RA). These diseases exhibit phenotypic similarities that reflect premature biological aging (58). Correspondingly, features of cellular aging such as telomere attrition and accumulation of p16 positive senescent cells, are characteristic of CKD and other conditions within the diseasome of aging (8, 29). Unsurprisingly therefore, uremic inflammation resembles the systemic inflammation associated with aging effects on the immune system, termed “inflammageing” and similar pathogenic mechanisms have been observed in aging and CKD, specifically premature immunological aging (65). Associated with this is immunosenescence, which arises due to the chronic depletion of naïve T cells, B cells, and dendritic cells, an expansion of memory T cells, particularly pro-inflammatory CD4+CD28–T cells, along with impaired neutrophilic phagocytic capacity and the loss of reno-protective factors, including Klotho and bone-morphogenic proteins, vascular rarefaction, and increased oxidative stress (58, 66). Reactive oxygen species (ROS) levels can also regulate signaling via the stress activated kinases, p38, and JNK. These pathways are often activated in aged tissues and contribute to inflammation and cellular senescence (67–70).

Along with systemic oxidative stress, mitochondrial dysfunction is a partner in crime, that links together with inflammation and the effects of uremic toxins to accelerate the aging process (44, 58, 71, 72). Two protein-bound uremic toxins, indoxyl sulfate (IS), and p-cresyl sulfate (pCS), not removed by conventional dialysis, are potent inducers of inflammation, oxidative stress, and vascular endothelial cell injury (73) and contribute to both the progression of renal impairment (74) and associated co-morbidities (75). Advanced Glycation End Products (AGE), which are also uremic toxins, accumulate in CKD, leading to mitochondrial dysfunction, elevated ROS production and structural changes through macro-molecular cross-linking. Consequently, the AGE axis induces renal cytosolic oxidative stress and inflammation (76, 77) leading to cell and tissue damage and the induction of premature senescence in proximal tubular epithelial cells and mesangial cells *in vivo* (78, 79).

A hallmark of uremic inflammation is the activation and defective regulation of the innate immune system. Abnormal activation of the innate immune system, especially the increased activation of monocytes, contributes to systemic inflammation via increased synthesis of pro-inflammatory cytokines. Several circulating pro-inflammatory markers have been reported to gradually alter in CKD as renal function fails, including IL-6, fetuin A, and TNF, which have previously been associated with renal aging and disease (80). Activation of inflammasome signaling by cytokines, ROS, and Damage-associated molecular patterns (DAMPs) results in an increase in Il-1β and IL-18, leading to an imbalance in the regulation of epigenetic mediators of aging such as miRNAs. For example oxidative stress has been shown to suppress Klotho expression in HK-2 cells via the induction of miR-200c (81). Deregulated TGFβ has also been shown to epigenetically induce Klotho deficiency and renal fibrosis in mice via induction of a number of miRNAs, including miR-21, miR-192, miR-491, miR-382, miR-377, miR-214, and miR-433 (82). The resulting imbalance of pro- and anti-inflammatory macrophages, and mitochondrial damage leads to the chronic systemic uraemic inflammation associated with CKD (44). Additionally, a consequential increase in synthesis of pro-inflammatory cytokines and chemokines by senescent cells, as part of the senescence-associated secretory phenotype (SASP), exacerbates both the inflammatory burden and dysregulated aging processes in CKD (44).

## Klotho in CKD

The phenotypic characteristics of genetic Klotho deficiency, such as bone disease, VC, CVD increased FGF23 levels, hyperphosphatemia, and premature mortality, resembles the uremic accelerated aging phenotype in man (58, 83). In keeping with these observations, Klotho deficiency in CKD has been reported to enhance renal tubule and vascular cell senescence leading to defective endothelial function and impaired vasculogenesis (84). Current literature supports such a thesis, and has indicated that the development and progression of CKD is significantly associated with a dysregulated FGF23-Klotho pathway, resulting in hyperphosphatemia and endothelial dysfunction (8, 18, 53–55). Phosphate retention, progressive hyperphosphatemia, rising FGF23 levels and low Klotho expression are all observed in patients with progressive CKD and are associated with age-associated CVD. Correspondingly, more recent studies have reinforced these observations and shown that the development and progression of CKD associates with a decline in Klotho in animal models (18, 53–55, 85–87). Kidney specific Klotho deficiency has been observed to reproduce the phenotype of Klotho deficient animals, displaying very low circulating Klotho and high circulating FGF23 levels (88). Specific deletion of Klotho in distal tubules has also been reported to inhibit the increase in renal Pi excretion in response to FGF23 (89), which suggests that Klotho deficiency limits its regulation of FGF23 production and that hyperphosphataemia, which usually become evident in CKD stage 3, remains the principal regulator of FGF23 secretion in CKD (90).

Dysregulation of the FGF23—α-Klotho network already occurs at earlier CKD stages, where abnormal levels of calcium and phosphate, inflammation, increased apoptosis, and depletion of calcification inhibitors results in the promotion of an active calcification process (91, 92). As nephron numbers decrease with age and renal damage, there is a resultant increase in Pi excretion per nephron via up-regulation of FGF23 observed in all CKD patients, causing a lowering of active vitamin D levels in the serum followed by an increase in PTH (5, 8, 44, 93, 94).

Recent research has indicated that Klotho may be involved in the manifestation of comorbidities associated with CKD. Klotho, for example, is expressed in the choroid plexus of the brain and controls the brain-immune system interface in the choroid plexus (21). As the uremic phenotype is associated by behavioral impairments, such as dementia, depression and cognitive deficits (95) and Klotho enhances oligodendrocyte maturation and myelination of the CNS (96), the low Klotho state of CKD may contribute to the common manifestations of central nervous system dysfunction in CKD. The specific role of the low Klotho state in the complicated scenario of uremic cognitive dysfunction (97) and disruption of the blood-brain barrier when renal function declines (98) deserves further studies.

Hypertension, an almost ubiquitous feature of CKD that contributes to both progression of CKD and CVD, may in part be related to Klotho deficiency. Mice deficient in Klotho have been reported to develop salt-sensitive hypertension after high sodium challenge (99) and a Klotho single nucleotide polymorphism with salt-sensitive hypertension has been described in adults with newly diagnosed hypertension (100).

In the uremic milieu lower Klotho levels may be exacerbated by the accumulation of protein bound uremic toxins, that epigenetically dysregulate Klotho gene expression and by an increase in TNF, which impairs Klotho protein expression (5, 29, 101). This relationship with inflammatory processes appears to be mutually reciprocal. Klotho modulates anti-inflammatory response in the kidney via a NF-κB mediated pathway, while Klotho expression in turn, is down-regulated by pro-inflammatory cytokines, such as TWEAK, again via an NF-κB dependent mechanism. Thus, while NF-κB *per se* contributes to the regulation of Klotho expression (102), Klotho however, in turn down-regulates NF-κB (103) and inhibition of NF-κB has been shown to reduce expression of several pro-inflammatory cytokines and renal injury (104–106). Correspondingly, in keeping with this thesis, Klotho depletion has also been shown to contribute to increased inflammation in the kidney in a pre-clinical murine model (107).

### Klotho and Vascular Biology

Numerous studies have suggested that Klotho is critical for vascular health and its therapeutic administration in CKD can exert vasculo-protective effects. Vascular calcification develops early in CKD and is associated with a decline in kidney function and an up-regulation of Pi resulting in a high risk of cardiovascular mortality and morbidity (8, 108–110).

Accordingly, Klotho deficiency in mice with CKD is paralleled by the development of VC and the occurrence of high Pi levels. As over-expression of Klotho enhances phosphaturia and improves renal function, it is suggestive that dysregulation of Klotho function is a key element in the development of VC (27, 55).

Zhao et al. (111) have demonstrated that up-regulation of Klotho expression protects against VC in CKD, via the inhibition of mTOR signaling in VSMC, in keeping with CKD as a disease of accelerated aging (111, 112). Accordingly, the stable delivery of soluble Klotho has been reported to reduce chronic hyperphosphatemia and VC both *in vitro* and *in vivo* (113). In support of these observations, soluble Klotho has been demonstrated to directly suppress Pi uptake and mineralization induced by high Pi *in vivo*, whilst administration of recombinant Klotho increased soluble Klotho by vitamin D receptor agonists (VDRA) treatment. Accordingly, the stable delivery of soluble Klotho has been reported to reduce chronic hyperphosphatemia and VC both *in vitro* and *in vivo* (113). This is in keeping with data from other approaches to mitigate the effects of hyperphosphatemia driven VC. Lau et al., have demonstrated that in a mouse model of chronic kidney disease where dietary phosphate loading induced aortic medial calcification, administration of vitamin D receptor agonists (calcitriol or paricalcitol) reduced aortic calcification and increased the expression of the anti-calcification factor, osteopontin, in aortic medial cells (27, 114).

CKD-mineral Bone disorder (CKD-MBD) and renal fibrosis have both been associated with diminished Klotho expression. Studies in a mouse model of CKD-MBD have indicated that loss of Klotho expression is a key event in renal and bone injuries and that restoration of Klotho expression attenuated CKD related bone defects (115). These observations are supported by enhanced Klotho expression via differential promoter demethylation, which has been demonstrated to protect against kidney and bone injuries in a mouse CKD model (116). Renal fibrosis, is also affected by Klotho expression levels. Renal fibrosis occurs following incomplete healing of wounded kidney tissue in the course of CKD, after chronic sustained injury. It is characterized by glomerulosclerosis, tubular atrophy, and interstitial fibrosis (117). A reduction in Klotho can both be a result and cause of renal fibrosis and can promote a vicious circle of RF generation and mitigation (118). A key regulator of this renal fibrosis cycle in CKD is TGF-β, which stimulates the accumulation of matrix proteins to induce ECM, inhibition of matrix degradation and the regulation of myofibroblast activation (119). In keeping with this thesis, murine models have indicated that inhibition of Klotho increases TGF-β expression and that TGF-β subsequently reduces Klotho expression via a negative feedback loop (120). Induction of Klotho also directly mitigates the effects of other pro-fibrotic factors, such as Angiotensin II (Ang II) and basic Fibroblast growth factor 2 (bFGF2). Klotho has been reported to mitigate Ang II-induced renal damage and suppress bFGF 2 expression *in vivo* (121) in keeping with a proposed reno-protective role for Klotho via the inhibition of multiple signaling pathways.

## Discussion

The weight of basic science, pre-clinical, and clinical evidence suggests that modulation of Klotho function is an exciting and viable target for therapeutic interventions. As such, it is a prime therapeutic target for intervention in a range of diseases within the diseasome of aging, including CKD (8, 122, 123). Given the role of Klotho in the biology of aging, such interventions also fall within the remit of senotherapies (i.e., therapeutic agents and strategies to specifically target and mitigate the deleterious effects of aspects of aging process).

As such, Klotho-mediated therapies may be classed as geroprotective (i.e. mitigating or inhibiting the effects of macromolecular damage leading to loss of cellular and physiological resilience and function, including the effects of the SASP), and senomorphic (i.e. suppression of senescence effects without cytotoxicity). It is important to note, however, that the context of their application and time in the life course applied are key elements for any success. While most basic and pre-clinical studies have focussed on mid- to end of life application, data from early life interventions are sparse and require a more robust evaluation. This is pertinent to the concept of antagonistic pleiotropy, as aging is a continuous process occurring across the life course, thus suggesting that the effects of any interventions will be dependent on the context of the stage of the life course at which they are applied. Cellular senescence is likely to be subject to antagonistic pleiotropy, as senescent cells are not desirable in young individuals, but are essentially anti-oncogenic in the old. The potential for any intervention to generate adverse effects, especially subtle epigenetic or cryptic effects, thus needs evaluated from this perspective. Initial evidence, however, suggests that most interventions appear to be well-tolerated and have some beneficial effective. Indeed, over-expression of α-Klotho has been reported to reverse the phenotype and extended life-span in Klotho-deficient mice (11, 114, 124).

**Figure 2:**
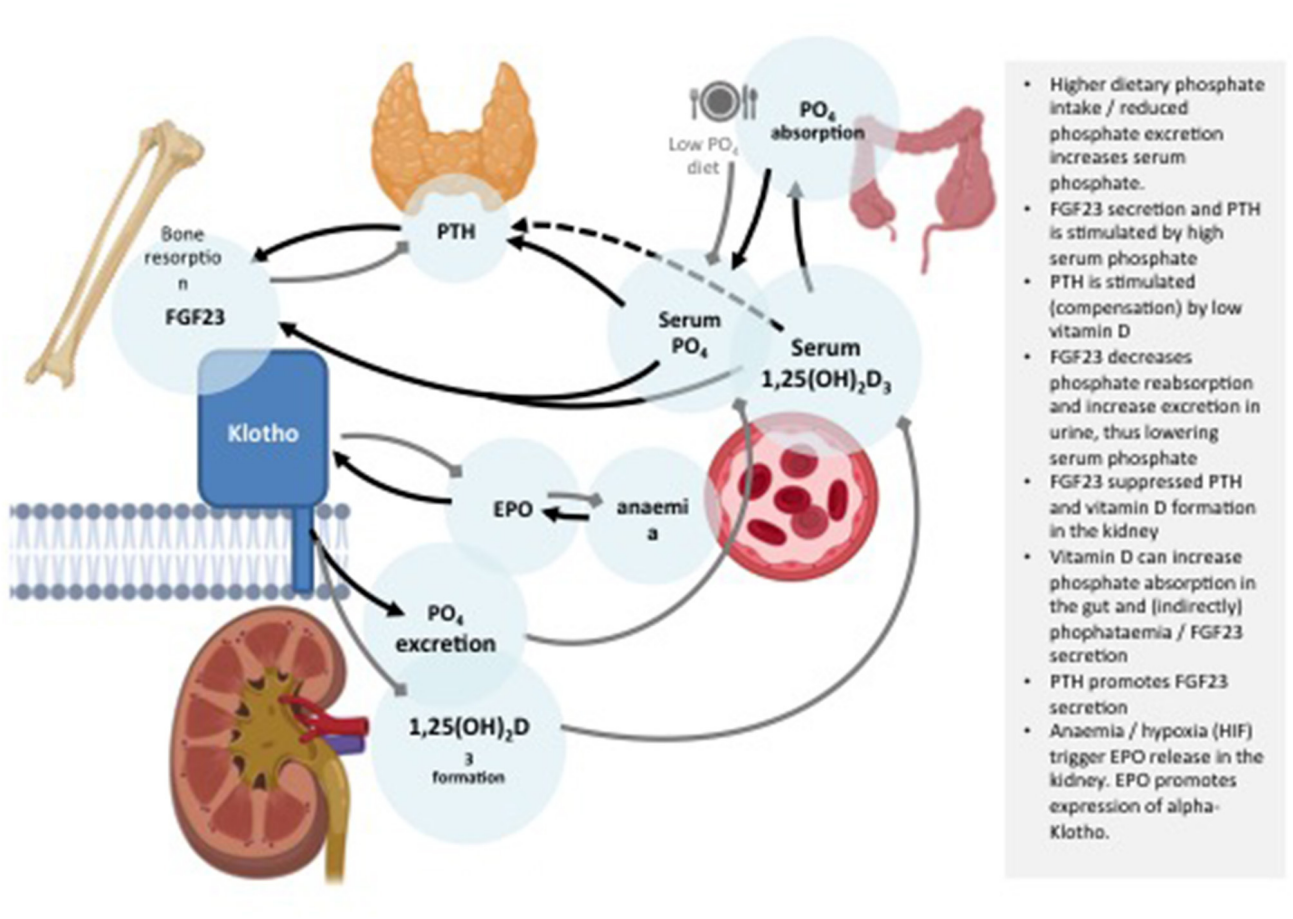
Schematic representation of a range of possible and existing Klotho based senotherapies.

Targeting Klotho in CKD is particularly pertinent on a number of fronts, Firstly, accurate detection of CKD at an early stage has proven problematic and currently no biomarkers are available which are suitably accurate, easily measured and sensitive enough (125). Current diagnostics rely on a GFR and urinary albumin:creatine ratio (UACR) (88). Thus, a blind spot occurs in early stage CKD where the eGFR and albuminuria are not above abnormal thresholds although kidney damage is ongoing. Soluble Klotho starts to decline in early CKD (Stage 2) (20). The decrease in Klotho in the kidneys correlates positively with diminution of eGFR (126–129), suggesting the decrease in soluble Klotho may accurately mirror an eGFR decrease in patients. Thus, measuring Klotho levels may provide earlier diagnosis (130). Secondly, standardized functional assays to detect Klotho activity are still currently lacking and several commercially available antibodies have been reported as unspecific and to cross-react with other proteins (131, 132). Thirdly, a number of strategies have been attempted to modulate Klotho expression with the aim if clinically translating findings. These strategies are illustrated in Figure 2 and are now discussed.

### Restoration of Klotho Levels and Activity

#### Administration of Exogenous Klotho

Several studies have suggested that pharmaceutical replacement or supplementation of Klotho can improve complications associated with CKD (8). Pre-clinical studies have indicated that administration of soluble Klotho can reduce renal fibrosis (120), VC (133), and EMT (134). Administration of soluble Klotho has proven a safe and effective treatment for kidney injury and preservation of renal function in early clinical studies (20, 135), though subsequent use of recombinant Klotho has yielded equivocal results (136).

#### Reactivation of Endogenous Klotho

Activation of Klotho has been reported to reduce levels of oxidative stress (137), improve mitochondrial function (137), reduce renal fibrosis (138), and inflammatory burden (8). Additionally it has been reported to mitigate the effects of premature aging (137) and EVA (139). Several methods have been explored to increase Klotho expression. These include:

- Demethylation of the Klotho promoter: Azuma et al. have demonstrated the use of Azacytidine (Aza), a DNA methyltransferase inhibitor, to increase Klotho expression ~1.5- to 3-fold *in vitro* by modulation of Klotho promoter expression (140). While this is feasible the laboratory, its clinical translation is more challenging, given the broad spectrum activities of Aza.
- Deacylation: Inhibition of histone deacetylases using HDAC inhibitors, such as trichostatin A or valproic acid have been shown to reduce inhibition of Klotho expression by TNF/TWEAK signaling in the kidney (86).
- PPAR-c antagonism: The PPAR-γ agonists troglitazone and ciglitazone, have been shown to increase both Klotho mRNA and protein expression *in vitro*. This induction was blocked by PPAR-c antagonists or by small-interfering RNA-mediated gene silencing of PPAR-c, suggesting this is a PPAR-c-dependent mechanism (141).
  The angiotensin II type 1 (AT1) receptor is also important in the aging process. Activation of the AT1 receptor by AngII is implicated in the age-related developments of hypertension, diabetes, and kidney disease. Disruption of the AT1 receptor gene in mice extends life-span and lowers levels of oxidative stress (142). Angiotensin II-type I receptor agonists have been used to demonstrate that cyclosporin-induced renal injury decreased Klotho expression. Yoon et al. have demonstrated that the Inhibition of the renin–angiotensin system (RAS) with Losartan restored Klotho expression in chronic CsA nephropathy (143). RAAS-blockers which block angiotensin/aldosterone have also been reported to reverse diminution of Klotho expression in rodents (5, 20, 144).
- Vitamin D derivatives e.g. paricalcitol: which has been shown to induce Klotho expression in rodent CKD models (145, 146). Another is Intermedin—a member of calcitonin family, which is thought to play a role in angiogenesis (147). Thiazolidinedione pioglitazone (designed to reduce insulin resistance by increasing peripheral glucose disposal and decreasing glucose production) resulted in increased expression of Klotho and protection against renal injury in aging (8). Statins have also been shown to induce Klotho mRNA expression (8).
- Exogenous recombinant peptide has also been used to raise soluble α-Klotho concentrations (20, 148) and supplementation with the peptide has been shown to attenuate LPS-induced kidney injury in mice (134). Numerous studies have shown that similarly to animals overexpressing α-Klotho, administration of the peptide also improved renal fibrosis in UUO and bilateral IRI models (10, 120, 121, 135, 148, 149), cardiac fibrosis in mice following induced injury (10), and could reduce acute to chronic disease progression with development of post AKI-cardiomyopathy. It was also effective in models of established injury.

### FGF23 Blockade

Blockade of FGF23 using Burosumab, has been demonstrated to be effective in control and improvement of rickets in patients with X-linked hypophosphataemia (150, 151). Use in CKD would require a very narrow and defined window of therapy to maintain phosphate balance. As FGF23 blockade in animal models resulted in worsened mineral balance and subsequent vascular abnormalities (152), more understanding of the FGF23-phosphate balance required are needed.

### Exercise

Numerous studies have demonstrated the effectiveness of exercise to prevent, reverse, or attenuate premature aging (58, 153, 154). Exercise is associated with regenerative tissue response (155), reduced atherosclerosis (156), and type-2 diabetes risk (157). Specifically higher Klotho concentrations are associated with increased lower limb strength (158), a reduced likelihood of developing Alzheimers (14), resistance to oxidative stress (159), and a lower risk of CVD and mortality (160). In pre-clinical murine models, s-Klotho levels have been associated with muscle strength and endurance capacity (161). However, the response to exercise was shown to differ with age (162) and may be subject to antagonistic pleiotropy. In humans, however, a long-term exercise programme resulted in increased soluble Klotho levels being observed in both young and old participants (162). Levels of soluble Klotho were also shown to be higher in trained young and old subjects compared to untrained subjects (163), and aerobic exercise was reported to increase plasma Klotho levels in young subjects (164). However, in contrast to these reports despite a functional improvement during a rehabilitation program, Klotho levels have been reported to remain unchanged in COPD patients (165). Several components of physical fitness have been related to related to s-Klotho plasma levels in the InCHIANTI study, which was a cohort survey initiated in 1998, primarily to study risk factors and mechanisms of mobility loss and aging (158, 166, 167). Evidence from general population cohort studies on the role of beta or gamma Klotho remains pauce. Optimal and comprehensive therapeutics strategies targeting Klotho might therefore still need to ensure address of each of individual isoforms to identify most the clinically beneficial isoform relative to a particular life course or disease contex. The concentration of circulating Klotho required, and that of each of its isoforms, will need to be precisely identified and genetic variability taken into consideration. High levels of Klotho could result in hyperparathyroidism and hypophosphatemic rickets (168), or have other toxic effects. Timing of Klotho delivery is also critical as soluble Klotho has been shown to be highly unstable in the blood and urine (169) and prevention of degradation may be essential.

Numerous compounds have been approved for use in CKD but with only some positive outcomes seen suggesting the extent of reactivating and or replacing Klotho and its effects are yet to be clarified (170).

### Nutrition

A number of studies have intimated that Klotho expression is amenable to nutritional intervention. Age-related changes in Klotho gene expression, particularly renal Klotho expression, and vitamin D metabolism can be affected by dietary Pi intake. As such, nutritional acquisition of Pi can radically affect age-related health, which opens up an easy avenue for therapy. There is good rationale for such an approach, as Pi has been linked to accelerated biological aging (including shortened telomeres, age related genomic hypomethylation) and poorer renal function (171) Higher all-cause and cardiovascular mortality risk have also been associated with Pi intake (172), and to accelerated vascular aging in the general population (60, 173). Additionally, in pre-clinical studies, high dietary Pi intake has been demonstrated to shorten life span in Klotho-deficient mice via activation of the AKT/mammalian target of rapamycin complex 1 (mTORC1) (174). Modulation of phosphate in the diet can be problematic. High protein foods tend to be high in phosphorus, and low phosphate diets present adherence challenges. Defining diets acceptable for patients is therefore an important focus. In a short phosphate restriction trial in patients with ESKD on hemodialysis, Tsai et al. have compared low and very low phosphate diets (phosphate:protein ratio 8 vs. 10 mg/g, 2 days each, separated by 5 days washout). While both diets led to a decrease in phosphate level, which was more marked with the very low phosphate diet, no difference was noted in FGF23 levels between the two diets, which performed similarly in decreasing FGF23. Supplementation with ketoacid analogs (KA, analogs of amino acids without the amino group, converted to AA without additional nitrogen) has been proposed to mitigate the risk of nutritional disorders associated with very-low protein diets (VLDP). In a short crossover study in CKD patients (1 week on very low-protein diet 0.3 g/kg/d supplemented with ketoanalogues; 1 week on low-protein diet 0.6 g/kg/d), Di Iorio et al. (175) have demonstrated a ~34% decrease in FGF-23 following the VLPD + ketoanalogues. In patients with CKD 3b-4, a 14-months low protein diets (0.6–0.8 g protein/kg/d) was effective in decreasing BMI, at the expense of muscle mass, with no noted reduction on serum phosphate. In comparison, ketoacid analog supplementation of the LPD was effective to maintaining FGF23 and serum Klotho levels, while these increased and decreased, respectively, in absence of KA (176). After 14-month of ketoacid-supplemented LPD supplemented valvular calcification score was also lower compared to patients following the LPD diet only.

Other pre-clinical studies have indicated that a high fat diet (HFD) as a surrogate for an energy rich and calorie dense western diet, results in age-related alterations in renal α-Klotho expression that could affect the responsiveness of dietary phosphate to vitamin D metabolism (177).

Alternative nutritional interventions have been designed to modulate Klotho-FGF23 inter-activity in CKD and thus address significant features associated with its pathophysiology including hyperphosphatemia, iron deficiency, and anemia [reviewed by (178)]. Interventions using iron therapies have displayed varied impact on Pi toxicity (179), though strategies involving iron-based phosphate binders do address the dual challenges of mitigating the effects of hyperphosphatemia and iron deficiency. In mice, ferric citrate administration (5% ferric citrate enriched diet) has led to phosphate binding, correction of iron deficiency, reduced circulating FGF23, and improved CKD outcomes (180).

Recently, use of a high magnesium diet has been demonstrated to prevent extensive vascular calcification in Klotho knock-out mice, opening up a novel route to tackling clinical VC. These effects were potentially mediated by reduction of inflammatory and extracellular matrix remodeling pathways within the aorta. Notably, serum parathyroid hormone, 1,25-dihydroxyvitamin D_3_ and calcium levels were unaffected. However, the high magnesium reduced bone mineral density and presented treated animals with possible osteomalacia (181).

Use of magnesium in this context is not unsurprising, given that Mg^2+^ is a key factor in the regulation of telomere biology and its level in the body is diminished in many diseases of aging (182).

Poor quality diets have been linked with higher level of dietary phosphate additives, higher fat intake, low vitamin D status, and lower bioactive-rich fruit and vegetables.

There is a rapidly emerging link between diet and FGF23, with associations described between diet quality/food (in)security and FG23 levels. In young adults studied in the context of coronary artery risk development, this has been tested under the hypothesis that food insecurity would be associated with intake of food and drinks with higher levels of phosphate additives, with potential impact on increased levels of FGF23 as a compensatory response to higher dietary phosphate intake (183). Pool et al. (183) have shown that individuals transitioning toward lower food security (i.e., “developing food insecurity”- defined as being unable to secure food of sufficient quantity and quality), was associated with increased odds of high FGF23 expression levels. This study was however unable to associate phosphorus intake with odds of elevated FGF23 expression in the cohort, in the absence of chronic kidney disease. Other pre-clinical studies have indicated that a high fat diet (HFD), as a surrogate for an energy rich and calorie dense Western diet, results in strong stimulation of murine FGF23 via inflammatory pathways, specifically TNFα formation (184). Correspondingly, Klotho deficiency in mice has been shown to accelerate and exacerbate HFD-induced arterial stiffening and hypertension, via down-regulation of vascular AMPKα expression, and activity (185). Yoshikawa et al. have also shown an age-related decrease in renal α-Klotho expression in mice, itself negatively associated with the vitamin D level response to a high phosphate diet (177).

Secondary analysis of the placebo-controlled Styrian Vitamin D Hypertension trial (186) trial in 181 adults with arterial hypertension and a low serum concentration of 25(OH)D (<30 ng/mL) has indicated that while vitamin D supplementation (2,800 IU daily) had no effect on FGF23 levels in the overall cohort, it did have an effect in subgroups with a baseline below 20 nl/L. The authors proposed that this notable rise in FGF23 levels in the low baseline subgroup could be explained by pronounced elevation of calcitriol levels and concomitant increased intestinal phosphate absorption. Systematic reviewing by Charoenngam et al. (187) has highlighted that the evidence supporting an impact of Vitamin D on FGF23 is strongest when studies measure intact FGF23 (instead of c-terminal FGF23) with a pooled standardized mean difference (SMD) of 0.36 (95%CI, 0.14, 0.57; *p* = 0.001; *I*^2^ of 36%) in vitamin D deficient individuals.

In a small sample of middle-aged sedentary adults (*n* = 74) enrolled in the FIT-AGEING study, a negative association has been demonstrated between total alcoholic intake and S-Klotho plasma levels (β = −17.031; *R*^2^ = 0.096, *P* = 0.013), even when adjusted for body composition parameters (188). Pre-clinical models have suggested a role for dietary bio-actives in Klotho expression. Specifically, Hsu et al. suggest that this can be achieved with resveratrol administration *in vitro* (NRK-52E cells) and *in vivo* (mouse kidney) with involvement of the ATF3/c-Jun pathway (189). However, subsequent analysis in the FIT-AGEING cohort was only able to show a weak association between the Dietary Inflammatory Index (DII) (190) or adherence to the Mediterranean Diet (191) with soluble Klotho. These associations disappeared when corrected for Lean Mass Index. Of particular interest is the role of dietary fiber in the prevention of renal function decline in CKD patients. With a low fruit and vegetable intake, fiber, and polyphenolic intake is typically reduced, which could have a direct impact on the functionality of the gut microbiota. In a randomized control trial in 52 patients with CKD (stages 3–5), supplementation with 50 g oats daily (rich in the soluble fiber beta-glucan and polyphenolics) for 8 weeks, did have a beneficial impact on serum potassium, but not Klotho levels or parathyroid hormone (192). There is a lack of high quality trials with well-defined inflammatory, iron and vitamin D status focusing on dietary bioactive intake administrated in food or supplemental forms, and new evidence in this field will facilitate improved dietary guidance for kidney health and healthy aging.

### The Gut Microbiota

The gut microbiota has emerged as a significant contributory factor to age related health [reviewed in (35)], where it appears to have a significant effect on the epigenetic landscape of aging. An understanding of any relationship between the gut microbiota and Klotho remains embryonic. However, there is an emerging indirect linkage based on the modulation of cell stress responses via processing of dietary acquired (poly)phenolic acids, which are potent Nrf2 agonists (193). The relationship between the microbiota and Nrf2 expression is an emerging therapeutic axis for a range treatment of the diseasome of aging and thus pertinent to Klotho. The transcription factor Nrf 2 controls a battery of over 390 cytoprotective genes and its expression is diminished with increasing age. Numerous nutritional intervention strategies are already in trial to enhance its age-related expression [reviewed in (32, 194)]. Furthermore, pre-clinical studies are supportive on such an approach to tackle CKD, via indirect up-regulation of Klotho achieved via Nrf2 agonism. Indeed, use of a synthetic Nrf2 agonist has already been demonstrated to induce Klotho expression and mitigate the effects of CKD in mice. Klotho *per se* (i.e., direct Klotho agonism) has not featured as a mediator of such effects, though this is intuitive with respect to β-Klotho, as its FGF19 substrate is secreted by bile acids produced by gut microbiota (5).

There is also an emerging relationship between the gut microbiota and the vitamin D/FGF23 axis. In murine models, Bora et al. (195) have demonstrated that antibiotic treatment increased vitamin D status, which in turn increased FGF23 levels after 14 days. However, the treatment effect on vitamin D was partly independent from the microbiota (as shown in GF mice) and potentially indicative of a direct impact on absorption.

## Conclusions

The emerging field of Geroscience seeks to address the cellular and molecular mechanisms underpinning the aging process and to understand how, when and why these become dysregulated, as the diseasome of aging manifests. Modulation of Klotho has emerged as a prime target for interventions within this space, having the potential to complement and synergise with other emerging therapies, including senolytics and live biotherapeutics, as well as more conventional pharmaceuticals. The ability to couple any emerging therapies with nutritional and exercise based approaches, offers a more holistic means of translating these to the general population and thus improving healthspan. Applications to age related diseases, such as CKD are exciting, and have already demonstrated clinical benefit with more promised to come.

## Author Contributions

All authors contributed to formulation, writing, discussion, and editing of the manuscript.

## Conflict of Interest

The authors declare that the research was conducted in the absence of any commercial or financial relationships that could be construed as a potential conflict of interest
